# Family‐Centred Care Rounds in a Neonatal Intensive Care Setting: An Implementation Sciences Feasibility Study

**DOI:** 10.1111/nicc.70276

**Published:** 2025-12-03

**Authors:** Adrien Saugy, Philippe Pythoud, Gwenaelle De Clifford‐Faugère, Zahra Rahmaty, Amandine Pereira Enes, Mirjam Schuler Barazzoni, Juliane Schneider, Carole Fletgen‐Richard, Magali Contino, Anne‐Sylvie Ramelet

**Affiliations:** ^1^ Institute of Higher Education and Research in Health Care University of Lausanne Lausanne Switzerland; ^2^ Department of Woman‐Mother‐Child, Neonatal Intensive Care Unit Lausanne University Hospital Lausanne Switzerland; ^3^ Haute Ecole Specialisée de Suisse Occidentale Institut et Haute Ecole de La Sante La Source Lausanne Switzerland

**Keywords:** family‐centred care round, parental stress, partnership, preterm infants, satisfaction

## Abstract

**Background:**

Parents of infants hospitalised in neonatal intensive care units (NICU) experience significant stress and require clear, consistent communication. Involving them in family‐centred rounds (FCR), a key component of family‐centred care (FCC), may help meet these needs.

**Aim:**

To assess the feasibility of implementing FCR in a NICU setting in Switzerland.

**Study Design:**

This one‐group pre‐post feasibility study was conducted from June to September 2022. Feasibility was evaluated using three dimensions: acceptability (participation rate and duration), implementation (fidelity of the intervention and open‐ended questions), as well as limited efficacy. Efficacy was assessed through parent‐reported stress and satisfaction (PSS, EMPATHIC‐N) before and after participation in FCR and healthcare professional (HCP) reported interprofessional collaboration (AITCS‐II) before and after implementation.

**Results:**

A total of 37 rounds were conducted with seven parents (24% participation). FCRs lasted on average 12m13s, compared to 8m49s for traditional rounds (mean difference: 3m24s). Fidelity, observed in 18 FCRs during the study period, showed some variability. Open‐ended survey questions from both parents and HCP provided qualitative insights on implementation determinants. Parents reported reduced stress after FCR and increased satisfaction in the ‘parental involvement’ subscale. Among professionals, only the ‘coordination of care’ subscale showed significant improvement.

**Conclusions:**

FCR implementation was feasible and showed potential to improve parental stress, satisfaction and interprofessional collaboration. Further evaluation of whether FCRs can be sustained over time is warranted.

**Relevance to Clinical Practice:**

FCR may enhance family partnerships in NICUs and improve care experiences for both families and healthcare teams, and promote a family‐centred care environment.

## Impact Statements

- What is known about the topic
  ○ The family‐centred care approach enables parents to be appropriately included in, and fully informed to make decision about their child's care and treatment during NICU hospitalisation.
  ○ Implementation sciences are an emerging field of research aiming to transfer research findings into practice, which is particularly relevant to nursing.
  ○ Family‐centred rounds remain inadequately implemented in care settings.
- What this paper adds
  ○ It is feasible to implement family‐centred rounds in the NICU.
  ○ Parents have expressed strong appreciation for family‐centred rounds.
  ○ Family‐centred rounds have the potential to improve interprofessional collaboration.

## 1 Introduction

The family‐centred care (FCC) approach ensures that parents are fully informed and included in decision‐making about their child's care and treatment during hospitalisation in intensive care. One effective way to improve communication and promote parental involvement in these decisions is through family‐centred rounds (FCR), which promotes partnership in care [1, 2]. However, implementing FCR in care units requires cultural adaptations in care delivery, making it a complex process that remains poorly documented [3].

## 2 Background

A neonate's hospitalisation in the Neonatal Intensive Care Unit (NICU) is extremely stressful for parents, supported by evidence that they experience higher rates of anxiety and post‐traumatic stress than the general population [4, 5, 6]. Effective communication with parents, along with clear and timely information delivery, was provided have shown to decrease parental anxiety and stress [7]. Parental anxiety and stress has also been shown to negatively impact the infant's health, particularly related to feeding, neurodevelopment and sleep [8, 9].

To improve the emotional well‐being of parents, FCC is internationally recommended [10]. This philosophy of care recognises the central role families play in the neonate's life [11, 12]. FCC promotes a partnership that is tailored to the unique needs and circumstances of each family across all health‐related systems and processes, including communication [1, 11, 13]. Allocating dedicated time for communication is essential, as the exchange of information and collaborative decision‐making are key components of FCC [13]. FCR provides a practical approach to implementing FCC in clinical settings [1, 14, 15]. During FCR, parents are systematically involved in sharing information, expertise and decisions related to the care plan [1, 11, 14]. The benefits of FCR, including improved communication and decreased parental anxiety have been well documented, especially in the paediatric intensive care unit (PICU) [11, 16]. Recommendations for effective FCR implementation include: (a) standardise the FCR process for efficiency [17, 18, 19, 20], (b) use clinical language that matches parents' levels of health literacy [16, 21, 22], (c) adapt FCR to each family according to their values and cultural specificities [11, 16, 22], (d) give families the freedom of choice to participate [13, 16, 18, 23], (e) ensure confidentiality [11, 24] and (f) create a suitable physical environment [19, 22, 24], preferably at the bedside [19, 23].

The benefits of FCR in the PICU setting have been demonstrated, but to a lesser extent in NICU settings [11, 16, 17]. However, FCR implementation needs to be adapted to the characteristics of the NICU context. Several barriers to implementation have been reported, including workflow changes, inadequate physical infrastructure and duration and efficiency of FCRs [11, 17]. Moreover, the modalities of FCR operationalisation are poorly documented in these studies. Limited understanding of the FCR contextualisation and its impact on outcomes hinders evidence transferability [17]. To promote FCR, it is essential to develop evidence on effective implementation processes and the feasibility of its implementation in complex care settings, such as the NICU.

## 3 Aim

To assess the feasibility of implementing the FCR in the NICU setting.

### 3.1 Specific Objectives

Feasibility was assessed based on three dimensions outlined in Bowen's framework: acceptability, limited efficacy and implementation criteria [25].

1. To evaluate the acceptability of the FCR for (1) healthcare professionals (time difference of FCR vs. traditional rounds) and (2) parental participation.
2. To explore the potential efficacy of the FCR with regard to (1) interprofessional collaboration and (2) parental stress and satisfaction.
3. To assess the implementation process (fidelity of the implementation and qualitative feedback).

## 4 Design and Methods

### 4.1 Design

A pre‐test post‐test feasibility study was carried out from June to September 2022.

### 4.2 Setting and Sample

This study took place in a 12‐bed level III NICU of a University Hospital in Switzerland with 850 medical and surgical admissions a year. The population consisted of healthcare professionals (HCP) and parents of NICU‐hospitalised patients.

### 4.3 Inclusion/Exclusion Criteria

#### 4.3.1 HCP

All HCPs working in the NICU were eligible for inclusion.

#### 4.3.2 Parents

Inclusion criteria were the following:

- Have a neonate hospitalised in the NICU. Parents of neonates hospitalised in the NICU for ≥ 5 days were recruited 2 days before FCR eligibility (from Day 7) to allow time for consent and baseline questionnaires.
- Be over 18 years.
- Speak and read French.

Exclusion criteria: parents of a clinically unstable neonate.

#### 4.3.3 Sample

A convenience sample of parents was recruited by two of the research team members (AS and PP). They were given written and oral information about the study and provided their electronic consent before completing the questionnaires. Each participant was assigned a code to ensure confidentiality. HCPs were invited to participate via their professional email address, while parents accessed a secure online platform (LimeSurvey) using a QR code and their anonymous code. Both groups completed the online questionnaires.

### 4.4 Intervention

FCR involved interdisciplinary rounds at the neonate's bedside with the family actively participating in the development of the care plan [14]. The core principles of the FCRs included the following: (1) conducting an interdisciplinary visit; (2) performing the rounds at the patient's bedside whenever possible; and (3) allowing for parental participation in discussions and decisions [14, 17]. The characteristics of our FCR are largely based on those of the traditional rounds at the participating hospital. The process was as follows: The rounds take place in the morning at the nurses' desk, and involved a physician, nurses and sometimes, other specialists. The physician ask questions, defines the treatment plan and updates the medical prescription. Throughout this process, parents were encouraged to participate in the discussion and shared decision‐making.

The other characteristics of FCR for this study were determined by the research team through an iterative process of interdisciplinary reflection. The development process included several key steps: a literature review in collaboration with a medical librarian, an analysis of the local context (unpublished data) and multiple meetings with a steering committee of stakeholders (physicians, nurse managers and clinical nurse specialists). Prior to implementation, healthcare professionals were offered e‐learning education to familiarise themselves with the principles and procedures of FCR.

#### 4.4.1 Description of the FCR Intervention

##### 4.4.1.1 Who?

(1) Parents of neonates ≥ 7 days of life; (2) stable medical situation; (3) first medical discussion with physicians (covering the infant's condition, breastfeeding, nursing care and available resources) where FCR was later added; (4) consented to participate (voluntary participation with oral consent); (5) spoke fluentl French (used regularly).

##### 4.4.1.2 What?

The Family Integrated care (FICare) model guided the characterisation of the nursing role and levels of parental involvement [15]. FICare extends the principles of FCC by recognising families as integral members of the NICU care team and promoting their active involvement, including but not limited to participation in medical rounds The FCR provided parents an opportunity to participate in medical and nursing discussions, to review the last 24 h of their infant's clinical situation and to participate in the development of the daily care plan.

##### 4.4.1.3 When?

FCRs were conducted in the morning, like traditional medical rounds.

##### 4.4.1.4 Where?

FCR were performed at the infant's bedside, at the nursing desk or in the unit conference room. The location of the FCR was determined collaboratively by the healthcare team and the parents, considering their preferences as well as clinical and organisational constraints. This flexible approach aimed to maximise parental comfort and engagement while ensuring the feasibility of the rounds in each specific situation.

##### 4.4.1.5 Who Does What?

The physician on duty introduced the concept of the FCR at admission, as this practice is relatively new in Switzerland. Two days before the first FCR, nurses were responsible for inviting parents to participate in the FCR. Nurses also prepared parents by explaining the purpose of the FCR and how FCRs work. A second explanation was provided to parents immediately preceding each FCR. During the FCR, the physician on duty summarised consultants' opinions and outlined the therapeutic plan, including short‐, medium‐, and long‐term objectives. Nurses actively contributed to clinical discussions, highlighting relevant patient issues and proposing appropriate interventions. Throughout FCR, HCPs adapted their language to address parents' health literacy needs [26]. After each FCR, families were given the opportunity to provide feedback on their experience. Finally, nurses documented relevant information that was discussed during the FCR, as well as up‐to‐date clinical observations, and nursing assessments and diagnoses.

##### 4.4.1.6 How?

A modified I‐SBAR (Introduction, Situation, Background, Assessment, Recommendation), adding parents' evaluation of their child's clinical situation, has been created and was used to enable parents and HCPs to communicate their observations during FCR. There are four progressive stages of parental involvement during FCRs: (1) listening and introducing oneself, (2) sharing observations, (3) introducing the infant at the start of rounds and (4) giving a report of the infant's last 24 h using a guide. Parents were expected to be involved in at least stages (1) and (2), depending on their preferences and confidence level [15].

### 4.5 Variables

#### 4.5.1 Sociodemographic Data

Sociodemographic data from HCPs and parents were collected via a secured online platform, LimeSurvey. For HCPs, sex, age, profession, number of FCRs they participated in and years of practice in their profession and in a NICU were collected. For parents, sex, country of origin, age, level of education, employment status, having other children at home and maternal complications were documented. For the neonate, gestational age at birth, main medical diagnosis and duration of hospitalisation were recorded.

#### 4.5.2 Acceptability

The duration of both traditional rounds and FCRs was measured using a stopwatch from the beginning of the round to the time when the medical plan was finalised, and prescriptions were made. The participation rate (FCRs performed/FCRs eligible but not performed) and the times difference between traditional rounds and FCRs were recorded once a week during the 3 months of implementation.

#### 4.5.3 Limited Efficacy

##### 4.5.3.1 HCP

Interprofessional collaboration was measured using the Assessment of Interprofessional Team Collaboration Scale II (AITCS II), composed of 23 items measuring three domains: partnership (8 items), cooperation (8 items) and coordination (7 items) [27]. Each item is rated on a 5‐point Likert scale from ‘1 = never’, ‘2 = rarely’, ‘3 = occasionally’, ‘4=most of the time’ to ‘5 = always’. Mean score for each domain was calculated. Values below four indicate inadequate perception of the AITCS II domain [27]. The French version used in this study was translated and culturally adapted in a previous study (the OCTOPUS‐3 study, unpublished data). The AITCS II was completed in LimeSurvey in the month prior to implantation and 3–4 months post‐implantation.

##### 4.5.3.2 Parents

Participants completed parenting stress and satisfaction questionnaires before the first FCR they participated in and after the last FCR they participated in. Parental stress was measured with the 14‐item Perceived Stress Scale (PSS), which has been validated in several contexts [28]. Each item is rated on a five‐point Likert‐type scale from ‘1 = never’, ‘2 = almost never’, ‘3 = sometimes’, ‘4 = fairly often’ to ‘5 = very often’. The total score was obtained by summing the 14 items and dividing the score by the number of items. A higher total score implies higher stress. Adequate psychometrics properties were demonstrated with construct validity and internal consistency (fidelity, Cronbach's alpha between 0.72 and 0.99 depending on domain). The questionnaire French‐speaking version of the EMpowerment of PArents in THe Intensive care‐Neonatology (EMPATHIC–N) was used to evaluate parental satisfaction [29, 30]. The EMPATHIC–N is composed of 65 items measuring five domains: information, care and treatment, organisation, parental participation and professional attitude. Each item is rated on a 6‐point Likert scale from 1 ‘absolutely disagree’ to 6 ‘fully agree’. A total mean score ≥ 5 reflects parental satisfaction; a score below this cut‐off indicates the need for improvement in the quality of the care provided. This questionnaire was adapted to the NICU [29], and translated and validated in French [30] with adequate psychometrics properties (domain Cronbach's alpha between 0.73 and 0.92) [29, 30].

#### 4.5.4 Implementation

The fidelity of FCR execution was assessed weekly for 3 months post‐implementation using an observation grid developed by our team. This grid was adapted from the fidelity checklist used by Ho and al., with permission [31], and pre‐tested by the project's pre‐implementation resource group. It was further tailored internally, based on key characteristics defined by the group for use in the NICU context, although it has not yet been published. Four open‐ended questions assessing general satisfaction about FCRs and barriers to its implementation have been added to the HCP questionnaire: (1) Regarding FCRs, do you think that good interprofessional collaboration can have a positive impact on the quality of care and satisfaction of patients and their families?; (2) In your opinion, what are the advantages and disadvantages of including patients and their families in FCRs?; (3) In your opinion, what are the facilitators and barriers to include patients and their families in FCRs?; and (4) Have you ever included parents in a FCR? In addition, parents were asked to answer three open‐ended questions: (1) How did you feel during your participation in the FCR?; (2) What elements could have prevented or facilitated your participation in medical‐nursing visits?; and (3) What are your general impressions of communication with healthcare providers.

### 4.6 Statistical Analysis

Data were analysed using Stata 17.0 [32] and SPSS (IBM Statistics version 28) with a α = 0.05. Descriptive statistics were used for categorical variables (absolute and relative frequencies) and continuous variables (mean, standard deviation, median, interquartile range).

#### 4.6.1 HCP

A Chi squared test was performed to compare sociodemographic differences of HCP pre‐ and post‐implementation, as different HCPs were involved. Interprofessional collaboration (AITCS II) pre‐ and post‐implementation was assessed by an independent *t*‐test.

#### 4.6.2 Parents

PSS and EMPATHIC‐N data were analysed as dependent variables, and means, standard deviations and a Wilcoxon ranks test were calculated.

#### 4.6.3 Qualitative Data

Qualitative data were analysed using thematic analysis by two independent researchers (AS and PP) and verified by a third team member (ASR). Reflexive thematic analyses based on Braun and Clarke [33] and including inductive and deductive analyses guided by the Consolidated Framework for Implementation Research (CFIR) domains (intervention characteristics, individual characteristics, internal context, external context and implementation process) were used [34].

### 4.7 Ethics

This study does not fall within the scope of the Swiss Federal Act on Research involving Human Beings. Therefore, it was reviewed and approved (2 May 2022) by the Lausanne University Hospital institutional review board.

## 5 Results

### 5.1 Socio‐Demographic Data

#### 5.1.1 HCP

A total of 24 HCPs in the pre‐implantation phase and 20 in the post‐implantation were recruited out of the 99 HCPs working in this NICU (64 nurses and 35 physicians). Pre‐ and post‐implantation participants showed no significant differences in sociodemographic data (Table 1). Twelve participants (50%) had already performed FCRs in other units/departments pre‐implantation.

**TABLE 1:** Healthcare professionals' sociodemographic data.

Variables	Pre‐implementation (*n* = 24) *n* (%)	Post‐implementation (*n* = 20) *n* (%)	*p*
Sex			
Males	3 (12.5)	2 (10.0)	1.00 (*χ* ^2^ = 0.68)
Females	21 (87.5)	18 (90.0)	
Age (years)			
26–35	9 (37.5)	8 (40.0)	0.99 (*χ* ^2^ = 0.29)
36–45	10 (41.7)	8 (40.0)	
> 45	5 (20.8)	4 (20.0)	
Profession			
Nurse	4 (16.7)	6 (30.0)	0.94 (*χ* ^2^ = 2.91)
Expert NICU nurse	7 (29.2)	6 (30.0)	
Nurse trainer	2 (8.3)	5 (5.0)	
Nurse manager	1 (4.2)	1 (5.0)	
Advanced practice nurses (ICLS)	1 (4.2)	0 (0)	
Physician assistant	1 (4.2)	1 (4.2)	
Head of clinic	2 (8.3)	1 (4.2)	
Physician manager	5 (20.8)	4 (20.0)	
Other	1 (4.2)	0 (0)	
Number of FCR performed			
Never	12 (50)	2 (10.0)	0.02* (*χ* ^2^ = 12.13)
1–3	5 (20.8)	11 (55.0)	
4–6	4 (16.7)	4 (20.0)	
11–15	1 (4.2)	3 (15.0)	
> 16	2 (8.3)	0 (0)	
Years of practice			
< 5	2 (8.3)	3 (15)	0.90 (*χ* ^2^ = 603)
5–10	7 (29.2)	5 (25.0)	
11–15	3 (12.5)	3 (15.0)	
> 16	12 (50.0)	9 (45.0)	
Years of practice in the NICU			
< 5	7 (29.2)	7 (35.0)	0.81 (*χ* ^2^ = 0.98)
5–10	3 (12.5)	3 (15.0)	
11–15	8 (33.3)	4 (20.0)	
> 15	6 (25.0)	6 (30.0)	

Abbreviation: FCR, family‐centred round.

* *p* < 0.05.

#### 5.1.2 Parents

Among the 13 parents approached during recruitment, one refused to participate, three were not eligible and two did not complete the post‐intervention questionnaire. Complete data were collected from seven parents (five mothers and two fathers), all of whom were from European countries (see Table 2).

**TABLE 2:** Parents' sociodemographic data.

Variables	*n* (%) *n = 7*	Mean (SD) Min–Max
Parent		
Mother	5 (71)	
Father	2 (29)	
Age		
31–35	6 (86)	
36–40	1 (14)	
Cultural background		
European	7 (100)	
Education level		
Secondary education	1 (14)	
Higher education	3 (43)	
Apprenticeship	1 (14)	
Other	2 (29)	
Employment status		
Employed	6 (86)	
Not employed	1 (14)	
Children	2 (29)	
Yes	2 (29)	
No	5 (71)	
Delivery		
Vaginal delivery	1 (14)	
Caesarean section	6 (86)	
Complications	3 (43)	
Newborn		
Gestational age (weeks)		30.6 (4.8) 26–39
Length of hospitalisation (days)		10.1 (5.3) 5–21
Main diagnosis		
Prematurity	4 (57)	
Other diagnosis	3 (43)	
Previous NICU experience	1 (14)	

### 5.2 Acceptability

#### 5.2.1 Duration of FCR

Duration of 18 FCRs and 19 traditional rounds were recorded. The mean duration was 12.22 min (SD = 4.80 min) for FCRs and 8.82 min (SD = 7.73 min) for traditional medical rounds.

#### 5.2.2 Parental Participation Rates

**FIGURE 1:**
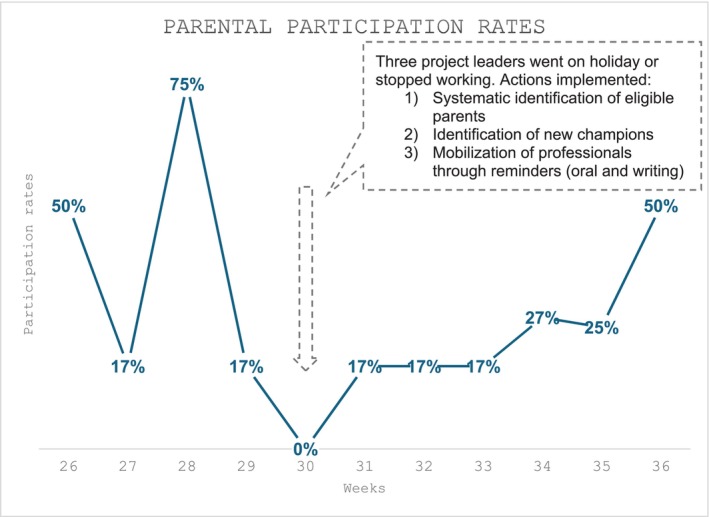
Parental participation rates.

Parental participation rates range from 0% to 75% on 37 occasions in 3 months post‐implantation (Figure 1). The main reasons for non‐participation were absence of parents, parents who were not informed of the study and medical unavailability (physicians who were either unavailable due to staffing shortages or they were attending medical call outside the unit).

### 5.3 Limited Efficacy

#### 5.3.1 HCP: Interprofessional Collaboration

No statistically significant difference in the total score was found between the pre‐implementation scores (Mean = 3.75, SD = 0.36) and post‐implementation scores (Mean = 3.52, SD = 0.49), as shown by a Student's *t*‐test (*p* = 0.08). Of the three subscales, coordination improved significantly (*p* = 0.04, see Table 3).

**TABLE 3:** Limited efficacy of healthcare professionals and parent‐reported outcomes.

Healthcare professionals outcomes	Pre‐FCR (*n* = 24)		Post‐FCR (*n* = 20)		Student's *t*‐test	
	Mean	SD	Mean	SD	Mean diff	*p*
AITCS subscales						
Partnership	3.54	0.49	3.76	0.50	0.22	0.15
Cooperation	3.90	0.46	4.00	0.37	0.10	0.44
Coordination	3.11	0.71	3.50	0.48	0.39	0.04*
Total AITCS II	3.52	0.49	3.75	0.36	0.23	0.08

Parent‐reported outcomes	Pre‐FCR (*n* = 7)		Post‐FCR (*n* = 7)		Wilcoxon ranks test	
	Mean	SD	Mean	SD	Mean diff	*p*
Total PSS	2.79	0.92	2.41	0.68	−0.38	0.123
EMPATHIC‐N subscales						
Information	4.66	0.49	4.77	0.34	0.11	0.285
Care treatment	4.87	0.21	4.92	0.11	0.05	0.715
Parental participation	4.71	0.31	4.79	0.28	0.08	0.046*
Organisation	4.73	0.20	4.75	0.26	0.02	0.915
Professional attitude	4.97	0.04	4.90	0.17	−0.07	0.109
Total EMPATHIC‐N	4.83	0.17	4.79	0.19	−0.04	0.753

Abbreviations: AITCS, Assessment of Interprofessional Team Collaboration Scale (mean AITCS > 4: good collaboration); EMPATHIC‐N, empowerment of parents in the intensive care neonatal; PSS, perceived stress scale; SD, standard deviation.

* *p* < 0.05.

#### 5.3.2 Parents

##### 5.3.2.1 Parents' Perceived Stress

There was no statistically significant difference in total perceived stress scores (*p* = 0.123, see Table 3).

##### 5.3.2.2 Parental Satisfaction

A statistically significant difference for the EMPATHIC‐N parental participation subscale (*p* = 0.046) was found between pre‐ and post‐intervention (see Table 3). For the total score, no significant difference was found (*p* = 0.753). The general satisfaction questions indicate that 100% of parents would recommend FCRs and participate in a future FCRs, if they had to return to the NICU.

### 5.4 Implementation

#### 5.4.1 Degree of Implementation

Mothers were present in 94% of FCRs, and both parents in 17%. Only one FCR was with the father alone (6%). The majority of FCRs were conducted at the nursing desk (72%), while 17% took place at the infant's bedside and 11% occurred in the conference room. During the majority of FCRs (72%), parents chose to share their observations. In 28% of cases, parents preferred to adopt a more passive role and simply listened to the discussions. Participation was voluntary, and parents were free to choose the level of involvement they felt most comfortable with during the FCR. On average, parents asked two questions during the rounds Confidentiality issues were reported in three FCRs conducted at the nursing desk and in two conducted in patient rooms, mainly due to the presence of other parents in close proximity. In the majority of FCRs, both parents and HCPs appeared not to be highly stressed, based on the observation grid (83% in both cases) or sought eye contact (89% and 94%, respectively). Language was adapted to parents in 83% of cases. Medical jargon, abbreviations, or complex terminology were used in 17% of the FCRs, which adversely affected parental understanding. Interruption occurred in 50% of FCRs. The modified I‐SBAR was used completely in 61% of cases.

#### 5.4.2 Qualitative Feedback

HCP: Post‐implementation, 20 HCP responded to the four open‐ended questions. Most participants were women (90%) and nurses (65%). Of the respondents, four (20%) were senior physicians and one (4.2%) was a senior nurse. Two participants (10%) had never participated in an FCR. Eleven participants (55%) had participated in between one and three FCRs, and seven (35%) in more than four FCRs. Our results, structured according to the CFIR framework, highlighted key factors influencing implementation (see Data S1). These factors included (a) the intervention being perceived as adaptable, not so advantageous and somewhat complex (intervention characteristics); (b) external pressure supporting its adoption (outer setting); (c) internal factors, such as structural characteristics, resource availability and the relative priority given to the project (inner setting); (d) individual beliefs, self‐efficacy, and readiness for change (characteristics of individuals); (e) active engagement and systematisation of information.

#### 5.4.3 Parents

Thematic analysis, as described by Braun and Clarke [33] of the qualitative data (*n* = 7) revealed two main themes: satisfaction and self‐efficacy.

1. Satisfaction encompasses three sub‐themes describing aspects of the parents' subjective experience of the NICU: the feeling of consideration (‘Be integrated in our child's recovery’ PRL 6160), the attitude of HCPs (‘The HCPss are (…) very attentive and empathetic, whether with the baby or the parents’ PRL 3326) and communication with them (‘Very good, very appropriate exchanges’ PRL 1238).
2. Self‐efficacy encompasses three sub‐themes: the perceived legitimacy of parental presence (‘Being present and having the opportunity to do so’ PRL 3486), preparation for FCRs (‘Understanding would be easier if we had the target values, or standards to refer to when values are stated’ PRL 0863), and the literacy of its content (‘For my husband, the very technical terms that come under the medical field are difficult to understand, whereas being a nurse it didn't cause me any problems’ PRL 9337).

Parents reported feeling integrated during FCRs. Participation in the FCRs appeared to enhance their awareness of personal preferences regarding involvement and information needs. However, several barriers remained that hindered their full engagement in the process. Their need for preparation was not always met. Language during FCRs was not always accessible, and participation levels and perceived legitimacy varied from one individual to another.

## 6 Discussion

This study aimed to assess the feasibility of implementing the FCR in a Swiss university hospital NICU. FCRs enhanced parental involvement in care, yet participation remained low overall and was primarily driven by mothers. Parents expressed strong appreciation for FCRs, reflecting their value in improving the family's care experience. For HCPs, implementing FCRs in the NICU setting proved to be feasible and acceptable, with the potential to improve interprofessional collaboration. The slightly longer duration of FCRs compared to traditional rounds was acceptable for HCPs, suggesting optimisation of comprehensive care and efficiency.

### 6.1 Acceptability

We observed that FCRs were more than 3 min longer than traditional rounds, which is comparable to other studies, which reported similar FCRs duration [18, 35] or slightly more time needed, increase of 1–3 min [22, 36, 37]. Given the differences between FCRs modalities, it is difficult to identify which factors act on the lengthening or shortening of the time of FCRs [17]. The duration of FCR is not determined by the time provided to the parent, but by the change in attitude of HCP in the presence of the parent [22]. The format of the FCRs in our study was identical to the traditional round. A change in format could have helped to maintain or reduce the duration of FRCs. For example, Lopez et al. have succeeded in reducing the duration of FCR by 34% by implementing a structured and interdisciplinary FCR, and by educating the whole team [38].

The average parental participation rate in our study was 24%, which corresponds to the lower range of parental participation rates reported elsewhere, namely between 17% and 94% (median rate 57.5%) [17]. A temporary drop in participation in week 30 coincided with the absence of key team members supporting implementation. Participation increased to 50% in the following weeks as additional professional leaders were engaged, eligibility identification procedures were standardised, and project communication was reinforced. Tripathi et al. [19] reported a similar trend regarding the evolution of participation rates after implementing the FCRs. Their participation rate rose from 0% to 43%, then fell to 28% before rising to 40% and stabilising at 38% [19]. These results illustrate that the success of FCR implementation is related to the human resources available and the leadership of the project's stakeholders.

Our qualitative results allow us to understand reasons for non‐participation: parents have erroneous beliefs about their self‐efficacy, leading to a feeling of illegitimacy to participate in FCRs. These results are corroborated by other studies highlighting the main barriers to parental participation, including low self‐efficacy, lack of information on FCRs, misunderstanding of its purpose and incompatibility of schedules [11, 16, 22]. Recommendations to further improve parental participation include restructuring communication during FCRs [18], providing explicit invitations to participate [39] and facilitating early discussions with parents on their desired level of participation [40].

### 6.2 Limited Efficacy

In our study, interprofessional collaboration was low overall in pre and post implementation, especially in partnership and coordination domains [27]. Of the three subscales, only coordination improved significantly, but remained low (less than the cut‐off of 4). Coordination reflects the ability to work together to achieve common goals [27] and the willingness and ability to share knowledge and skills [41]. Furthermore, qualitative studies have reported improved perceived interprofessional collaboration as a result of FCR implementation [36, 42]. Interprofessional collaboration is a complex phenomenon, influenced by many factors such as education, communication, respect and trust, stress levels, shared power and understanding of different professional roles [43, 44]. Future research on interprofessional collaboration and FCRs could investigate the influence of these different factors in the NICU.

Our results showed that after FCRs, parental stress tended to decrease, although the difference is small and not significant, likely due to our small sample size. One study specific to FCRs in NICUs also shows similar results with no difference in parental stress between parents who participated in FCRs and those who did not [40]. A qualitative study indicates that the way of conducting FCRs and making a connection with the families appears to influence emotional feelings, such as anxiety [45]. Families who were prepared on what to expect during rounds and who were supported in their involvement felt comfortable with the situation and had less anxiety [45]. In our study, the nurse was responsible for preparing parents for FCRs, but was not involved in the operationalisation of the rounds, or the promotion of parental empowerment. In this context, extended nursing support may have helped mitigate the barriers that limited parental access to FCRs.

Our results highlighted a significant difference in parental satisfaction after FCRs only for the EMPATHIC‐N involvement subscale, although all other subscales increased. In the scientific literature, FCRs are generally associated with slight improvement in satisfaction [1, 11, 39]. In our NICU, pre‐implementation results of general satisfaction were already high. It is thus more difficult to obtain a significant improvement of satisfaction associated with a specific intervention.

## 7 Limitations

The sample of parents was smaller than expected, due to a low‐eligible population during the study period. Therefore, further research with larger sample sizes is warranted. FCR observations were performed by four different HCPs. However, biases related to multiple observers were minimised with the use of an observation grid. Our results could be biased by a Hawthorne effect, where FCR participants might have tended to perform better when observed during the FCR. This can be seen as a methodological limitation, but also as a strength in an implementation project, as this effect maximises everyone's performance. Also, some professionals had already taken part in FCRs in other departments in the pre‐implementation phase, while others had never taken part in an FCR in the post‐implementation phase. The post‐implementation survey was distributed to all members of the medical and nursing team (*n* = 99), regardless of their direct involvement in FCR. Since the intervention aimed to engage the entire team, some respondents were unable to participate during the implementation period. This certainly had a negative impact on our results, diminishing the real effect if all the department's staff had responded in the post‐test.

## 8 Implications

The findings of this review highlight several important implications for practice and future research regarding the implementation and evolution of FCRs. Future research is needed to support the involvement of families in the design and conduct of FCRs [17]. A next step would be the development of a virtual FCR, which could improve the accessibility of FCRs and participation rates, mainly for partners who may have to return to work soon after the birth of their infant [11, 46]. In this sense, Yager et al. suggested that virtual FCRs could provide a means to improve parent–HCP communication [23]. Furthermore, Rosenthal et al. have identified that virtual FCRs have the potential to enhance parental involvement and satisfaction, improve breastfeeding rates, reduce length of hospital stay and decrease medical errors [46]. However, one may question whether confidentiality concerns persist in this new form of FCR. A recent implementation study showed that, although privacy was initially a concern, HCPs were able to maintain confidentiality through adaptive strategies [47]. The successful implementation of FCRs in the NICU of one department of a university hospital opens the possibility of extending it to other departments. For example, an implementation study on the transfer of FCRs to another unit (e.g., PICU, surgical unit) could be relevant. From this perspective, it may be worthwhile to create a national network to support FCC implementation processes [48].

## 9 Conclusion

This study highlighted the feasibility of FCRs in the NICU context and highlighted the potential to improve parental stress, satisfaction, and interprofessional collaboration. In the weeks following implementation, the rate of parental participation was low and highly variable. The reasons were identified, and recommendations were made to improve parental involvement. Parents reported decreased stress from pre‐ to post‐FCR which was not significant, probably due to sample size. This study showed high levels of parental satisfaction, although the satisfaction score was high at baseline. Consequently, the satisfaction with FCC care only improved for the parental involvement subscale. The subscale coordination of care of interprofessional collaboration increased post‐implementation, which seems congruent with the nature of the intervention. Overall, these findings support the continued integration and refinement of FCRs in standard practice, as a strategy to strengthen family‐centred care in the NICU.

This study has positive implications for neonatal intensive care nursing as FCRs remain underutilised in NICU settings and also may not be carried out effectively in such settings. To support the implementation of FCRs in NICU settings, further research is needed on whether the FCR approach is sustainable over the long term. FCRs may also enhance family partnerships in NICU settings and improve care experiences for both families and healthcare teams, and promote a family‐centred care environment.

## Author Contributions

**Adrien Saugy:** conceptualization, methodology, investigation, formal analysis, writing – review and editing. **Philippe Pythoud:** conceptualization, methodology, investigation, formal analysis, writing – review and editing. **Gwenaelle De Clifford‐Faugère:** writing – original draft preparation, visualization. **Zahra Rahmaty:** formal analysis, data curation. **Amandine Pereira Enes:** conceptualization, methodology, investigation. **Mirjam Schuler Barazzoni:** conceptualization, methodology. **Juliane Schneider:** conceptualization, methodology, investigation. **Carole Fletgen‐Richard:** conceptualization, methodology. **Magali Contino:** conceptualization, methodology, investigation. **Anne‐Sylvie Ramelet:** conceptualization, methodology, resources, writing – review and editing, supervision.

## Funding

The authors have nothing to report.

## Ethics Statement

This implementation project was approved by the institution Evaluation Commission for Survey and Research Request (CEDE Commission); as this project fell outside the scope of the Swiss Human Research Act, there is no registration number but written approval of this project is provided.

## Consent

Participants gave written consent.

## Conflicts of Interest

The authors declare no conflicts of interest.

## Supporting information


**Data S1:** Supporting Information.

## Data Availability

The data that support the findings of this study are available from the corresponding author upon reasonable request.
