# Supplementary material for: Family‐Centred Care Rounds in a Neonatal Intensive Care Setting: An Implementation Sciences Feasibility Study
Source: Nurs Crit Care. 2025 Dec 3;31(1):e70276. doi: 10.1111/nicc.70276 (PMC12675979; doi:10.1111/nicc.70276)
Supplement: Supplementary file 1 — Data S1: Supporting Information. [file NICC-31-0-s001.docx]

**Supplementary file 1**

**Table 1. Factors affecting implementation**

| **Themes (104)** | **Subthemes** | **Context in the NICU** | **Verbatim and informal discussions** |
| --- | --- | --- | --- |
| Intervention Characteristics | Complexity | Changes in culture, power relations and the role of parents | *Caregivers have a somewhat “historical” need to hold knowledge rather than share it (ID 48).* |
|  | Adaptability | FCR is time-consuming but can save time during the working day.  FCR is not suitable for sensitive discussions. | *FCRs slow down the visit (ID 41). The time it takes us to talk to parents about what they didn't understand at the visit (they don't dare ask the doctors again) (ID 51). Increased visit time, as we feel obliged to explain technical terms, figures, standards, etc.... so that the family understands what is being said (ID 60).*  *FCRs reduce communication time after the round (ID 44).*  *The major disadvantage, in my opinion, is that during the FCRs, the multidisciplinary team cannot address questions specific to the family (ID 52).* |
|  | Relative advantage | FICare stage 1 is not very helpful | *I've done 2 FCRs in the NICU. They seemed to me to be a little less contributory; because parents were spectators, there was less input to give, as the babies were still small (ID 57).* |
| Outer Settings | External pressure | Frequent requests for information on the subject. | Informal discussions with university hospitals in Switzerland and France. Involvement in a project at our hospital. |
| Inner Setting | Structural characteristics | Structural problems relating to confidentiality, noise, and ergonomics.  Better medical organization would promote FCR. | *The structure of the department (inadequate nursing office, too much ambient noise) is a barrier to FCRs, and there is no real confidentiality (ID 36).*  *A medical organization that would not be disturbed by emergencies during the visit would be a factor facilitating FCR (ID 53).* |
|  | Relative priority | Numerous projects in progress | Informal discussions on current projects (e.g., implementation of a new computer system, new measures to counter nosocomial infections, a systematic review project, standardized management according to patient categories). |
|  | Available ressources | Partial resumption of training and increased time for trainers. | Informal discussions with leaders and trainers |
| Characteristics of individuals | Knowledge and beliefs about the intervention | FCR increases social inequalities.  Parents' view of the nursing role has changed | *The more educated families, who are more likely to be present, and who in fact already receive more attention and information spontaneously, seem to be the ones who will have the most access to the partnership visit and benefit from it the most! (ID 48).*  *Families who have taken part in the medico-nursing visit often say that they now see the nursing role differently (ID 60).* |
|  | Self-efficacy | Parents' view of professional competence | *I'm afraid of not doing right by the families (bad information, fears...) (ID 44).* |
|  | Individual stage of change | FCRs are stressful for young healthcare professionals. | *Stressful the first time (ID 69)* |
| Process | Engagement | Interdisciplinary work | *Interprofessional training using simulations involving both physicians and nurses could facilitate implementation of the practice in the department (ID 60).* |
|  | Doing | Little systematization of information | Informal discussions |
